# Editorial: Advanced polymeric materials and nanomaterials enabled construction and application of biomedical microdevices

**DOI:** 10.3389/fbioe.2022.982431

**Published:** 2022-07-22

**Authors:** Chao Ma, Xiaowen Huang, Yaolei Wang, Shaofei Shen, Wenming Liu

**Affiliations:** ^1^ Department of Mechanical and Aerospace Engineering, New York University, Brooklyn, NY, United States; ^2^ Department of Biomedical Engineering, New York University, Brooklyn, NY, United States; ^3^ School of Bioengineering, Qilu University of Technology, Jinan, Shandong, China; ^4^ School of Life Sciences and Engineering, Southwest Jiaotong University, Chengdu, Sichuan, China; ^5^ Shanxi Key Lab for Modernization of TCVM, College of Life Science, Shanxi Agricultural University, Taigu, Shanxi, China; ^6^ School of Basic Medical Science, Central South University, Changsha, Hunan, China

**Keywords:** biomaterials, microdevice, microfluidics, 3D printing, drug delivery, bioimaging

Over the last decades, various biomedical devices, fabricated with protocols utilized in chip manufacturing or microelectromechanical systems, have been developed to solve the fundamental biological questions as well as to enable practical clinical applications. Specifically, the integration and adaption of new polymeric materials and nanomaterials have greatly advanced the design and fabrication of advanced biomedical device, broadening its application capabilities and scenarios, ranging from point-of-care diagnostics and drug discovery to bioimaging. However, the critical issues associated with the construction and application of biomedical device are still being explored to release its full potential to fulfil the aim of precision medicine. This editorial summarizes the contributions to the Frontiers Research topic “*Advanced Polymeric Materials and Nanomaterials Enabled Construction and Application of Biomedical Microdevices*”, which is established under the scope of Biomaterials section and listed under two participating journals, Frontiers in Bioengineering and Biotechnology and Frontiers in Materials.

Isolation of circulating tumor cell (CTC) helps to elucidate its metastasis role in solid tumor, yet it remains a technical challenge. To effectively enrich viable and high-purity CTCs, Li et al., developed a new double spiral microchannel-based microfluidic platform with integration of two-stage inertial focusing and particle deflection. As a proof-of-concept study, they were able to separate CTCs from 12 cancer patients’ blood samples, highlighting the potential of the double spiral microchannel for label-free liquid biopsy in clinical settings. Fabricating microfluidic devices with soft lithography has significantly improved the versatility and applicability of microfluidic technologies for biological studies. Yet, conventional designs of microchannels limit the volumetric throughput of microfluidic device, especially for liposomes preparation in drug delivery. To tackle this issue, Shan et al., applied a high-resolution projection micro stereolithography to 3D print a multi-layer microfluidic chip to realize a higher total flow rate with design of critical dimensions that is not possible with conventional devices. Notably, this 3D-printed chip enables an ultra-high volumetric preparation of nanoliposome at a control size.

Biomedical imaging is a powerful tool for dynamically visualizing biological processes and pathological progression within the human body. For example, photoacoustic tomography (PAT) approaches, based on the acoustic detection of optical absorption from endogenous chromophore and exogenous contrast agent in human tissue or organ, demonstrate a non-invasive way of observation and measurement. However, current PAT strategy is mostly based on a single-element transducer which limits its application scenarios with predefined detection bandwidth. To improve the sensitivity and capability of PAT, Luo et al., constructed a stack-layer dual-element ultrasonic transducer. Integration of this novel transducer results in a broad-bandwidth PAT that can achieve better visualization of vascular network as well as monitoring of blood oxygen saturation in target tissue. Moreover, acoustic-resolution photoacoustic/ultrasonic endoscopy usually utilizes a point-focused transducer, allowing for ultrasound detection only in the focal region. To improve the lateral resolution of detection, Pang et al., developed a line-focused transducer that generates a more uniform sound field in comparison to traditional point-focused transducers, resulting in enhanced signal intensity and signal-to-noise ratio. To enable dual-modality of magnetic resonance imaging and photoacoustic imaging, Li et al., fabricated a new probe with cobalt core/carbon shell–based nanoparticles which following intravenous injection into glioblastoma-bearing mice accumulate within the tumors. Such a nanoparticle probe can thus help magnetic resonance imaging promptly to identify lesion locations and photoacoustic imaging to acquire high-resolution image and quantitative information of the tumor. In addition, Wen et al., demonstrated the co-applicability of photoacoustic tomography and multispectral optoacoustic tomography for non-invasively detecting histology in a rabbit tracheal stenosis model. This study expands the possibility of combining various detection tools for biomedical imaging in a multiplex manner.

Rapid and *in situ* measurement of the effect of candidate agents and treatment combinations on brain pathophysiology is critical for developing effective therapeutics for neural disorders. Kim et al., developed a miniaturized implantable microdevice platform for simultaneous drug delivery and measurement of its therapeutic outcome in the native brain tissue. Using murine models of Alzheimer’s disease, they demonstrated that implantable microdevices can controllably release microdoses of drugs within a local brain region to achieve therapeutic responses.

To sum up, this Research Topic highlights the major achievements of fabrication of biomedical microdevices with new materials and methodologies and their successful biomedical applications. With the incessant efforts contributed from researchers in the biomedical field, we believe that novel technical breakthroughs will not only deepen our understanding of the mechanisms regulating human health and disease, but also help to develop novel diagnostic strategies and better clinical interventions.

